# The infantile neuroaxonal dystrophy rating scale (INAD-RS)

**DOI:** 10.1186/s13023-020-01479-5

**Published:** 2020-07-29

**Authors:** Paldeep S. Atwal, Mark Midei, Darius Adams, Alexander Fay, Frederic Heerinckx, Peter Milner

**Affiliations:** 1 Retrotope Inc., Los Altos, CA USA; 2 Atlantic Medical Group, Morris Township, NJ USA; 3 University of California San Francisco (UCSF), San Francisco, CA USA

**Keywords:** Clinical outcome assessment (COA), Infantile neuroaxonal dystrophy (INAD), Infantile neuroaxonal dystrophy rating scale (INAD-RS), PLA2G6-associated neurodegeneration (PLAN)

## Abstract

**Background:**

INAD is an autosomal recessive neurogenetic disorder caused by biallelic pathogenic variants in *PLA2G6*. The downstream enzyme, iPLA_2_, plays a critical role in cell membrane homeostasis by helping to regulate levels of phospholipids. The clinical presentation occurs between 6 months and 3 years with global developmental regression, hypotonia, and progressive spastic tetraparesis. Progression is often rapid, resulting in severe spasticity, visual impairment, and cognitive decline, with many children not surviving past the first decade of life. To date, no accepted tool for assessing the severity of INAD exists; other commonly used scales (e.g. CHOP-INTEND, Modified Ashworth, Hammersmith Functional Motor Scale) do not accurately gauge the current severity of INAD, nor are they sensitive/specific enough to monitor disease progression. Finally, these other scales are not appropriate, because they do not address the combination of CNS, peripheral nerve, and visual pathology that occurs in children with INAD.

**Methods:**

We have developed and validated a structured neurological examination for INAD (scored out of 80). The examination includes six main categories of pediatric developmental evaluation: 1) gross motor-and-truncal-stability skills, 2) fine motor skills, 3) bulbar function, 4) ocular function, 5) temporo-frontal function, and, 6) Functional evaluation of the autonomic nervous system. A cohort of patients diagnosed with INAD were followed prospectively to validate the score against disease severity and disease progression.

**Results:**

We show significant correlation between the total neurological assessment score and months since symptom onset with a statistically significant (*p* = 6.7 × 10^− 07^) correlation between assessment score and disease onset. As hypothesized, the coefficient of months-since-symptom-onset is strongly negative, indicating a negative correlation between total score and months since symptom onset.

**Conclusion:**

We have developed and validated a novel neurological assessment score in INAD that demonstrates strong correlation with disease severity and disease progression.

## Introduction

Infantile neuroaxonal dystrophy (INAD, NBIA2A; MIM# 256600) is a major subtype of *PLA2G6*-associated neurodegeneration (PLAN), a heterogenous group of clinical disorders with varying severity comprising INAD, atypical neuroaxonal dystrophy (NBIA2B; MIM# 610217) and adult-onset dystonia-parkinsonism (PARK14; MIM# 612953). PLAN is caused by biallelic pathogenic variants in *PLA2G6*, and the specific phenotype of PLAN is based on various clinical, genotype-phenotype, neurophysiologic, radiographic, and laboratory features [1, 2].

The onset of INAD typically occurs between 6 months and 3 years of age, commonly presenting with psychomotor regression, gait disturbance, truncal hypotonia, and in some patients, strabismus and nystagmus. The disease progresses into spastic tetraparesis with symmetric pyramidal signs, progressive cognitive decline, loss of vocalization, optic atrophy, and bulbar dysfunction. The progression is usually rapid and patients rarely survive beyond their first decade, even with supportive care [3–6]. Seizures may present early or late in the disease course but are reported in only a minority of patients [4, 6–9]. Histopathology may show the presence of axonal spheroid bodies in both the central and peripheral nervous system [6, 7, 10], and neuroimaging usually reveals pathognomonic inferior cerebellar atrophy; in some cases, iron accumulation is seen in the globus pallidus. Given its ultra-orphan status, no consensus guidelines exist on the management and treatment of INAD. With no approved therapies, the management of INAD remains supportive, with symptomatic treatment for issues such as contractures (e.g. physical therapy) and feeding difficulties (e.g. feeding support) the mainstay of treatment.

The status of INAD as an ultra-orphan disease has limited the ability to report on clinical observations in significant numbers of patients in any one study, and there is currently a lack of any meaningful measures of disease status or progression. To date, no accepted tool for assessing the severity of INAD exists; other commonly used scales (e.g. CHOP-INTEND, Modified Ashworth, Hammersmith Functional Motor Scale) do not accurately gauge the current severity of INAD, nor are they sensitive/specific enough to monitor disease progression. Finally, these other scales are not appropriate, because they do not address the combination of CNS, peripheral nerve, and visual pathology that occurs in children with INAD.

Although the clinical progression of INAD has been documented in prior natural history studies, these studies were retrospective and may or may not have captured the full spectrum of manifestations of INAD due to their retrospective nature or the patterns of decline [11]. This present prospective collection of 40 genetically confirmed cases represents the largest cohort of INAD patients reported to date, and thus is the most comprehensive cohort available to create a meaningful clinical outcome assessment (COA). A meaningful COA of INAD could serve as an accurate measure of natural history and progression, and further refine it from existing cohorts [12]. In addition, until disease-modifying therapies become available, these data have valuable confirmatory diagnostic and prognostic value for clinicians, patients, and families.

This report describes a clinical rating scale for INAD, INAD-RS, which takes into account the six main categories of pediatric developmental evaluation: 1) gross motor-and-truncal-stability skills, 2) fine motor skills, 3) bulbar function, 4) ocular function, 5) temporo-frontal function, and, 6) Functional evaluation of the autonomic nervous system. It is designed such that each category of efficacy measures how patients function in daily tasks and interactions. While most are function-oriented with clear outcomes on patient performance (e.g. gross motor, fine-motor), some, such as bulbar function, are critical to ADLs and vital functions such as feeding and survival. In aggregate, the items on INAD-RS directly assess a patient’s 1) ability to carry on daily life activities (function) including those critical for survival, 2) ability to interact with their environment and caregivers, and 3) symptomatic status related to how they feel.

## Materials and methods

### Ethical approval

An IRB-approved written informed consent was obtained from all patients and their parents enrolled in this study.

### Scale development

The first step we took was to collate and cross-examine expertise of key opinion leader (KOL) pediatric neurologists, which included some authors of this paper, the author’s medical expertise in neurogenetics (including US board-certifications in clinical genetics, medical biochemical genetics, pediatric neurology), and our own medical examinations of 40 INAD patients with a range of severity. Subsequently we created a standardized a scoring system of 40 specific assessment measures of either ADLs or vital functions that are abnormal in INAD, named INAD-RS. We also imported elements of age-appropriate (adjusted for disease state), clinically meaningful, validated, and standardized scales used in other pediatric neuro-degenerative diseases such as the Bayley Motor Scale. Details on adaptions describing the development of assessment measures are outlined in supplementary document 2 entitled ‘INAD Rating Scale Development Scoring Table’.

### Goals and limitations of INAD-RS

INAD-RS is not designed to diagnose INAD; rather, its purpose is to quantify disease status and disability, however, it may be of value in the early evaluation of an infant with regressive features to confirm the suspicion of INAD before MRI or molecular diagnostic confirmation. Given this purpose, it aims to include all areas of clinical impairment observed in INAD as outlined above. INAD-RS is designed primarily for pediatric neurologists as a routine part of clinical care and monitoring. The scale takes ~ 10 min to complete and requires minimal supportive apparatus (e.g. a small block and a toy to attract child’s attention). One part of the examination, assessment of optic pallor, may require an ophthalmologist’s input if indirect fundoscopy is not possible. The other parts of the ocular exam require familiarity with assessments of nystagmus and strabismus and the range of possible severity of these signs that can only be acquired through clinical acumen and experience.

INAD-RS is intended to serve as a global, convenient measure of INAD disease status and progression and could be used in early stages of interventional studies or as a meaningful measure of progression in individuals or groups.

### Subjects and scale administration

All patients examined had a molecularly confirmed diagnosis of INAD via identification of bi-allelic pathogenic variants in *PLA2G6.* Patients were evaluated across multiple centers, including two sites in North America, and individual sites in China, Egypt, India, and Tunisia. All examinations were videotaped and reviewed for inter-rater reliability (see section on Inter-rater reliability). All examiners received prior training on the scale by way of discussion with an experienced examiner, and/or a training manual (Supplementary Document 1), and/or observing prior videos of high, medium, low scoring patients.

The month and year of symptom onset was determined by detailed history taking. In most cases, the first symptoms were gross motor, such as delays in balance or walking and/or developmental regression. The date of symptom onset was recorded as month and year. The date of each visit was entered as month/day/year. In addition, year of birth and age at time of assessment were obtained from medical records and family reports.

### Scale content

We designed INAD-RS so that each category measures how the patients function in daily tasks and interactions. While most are function-oriented with clear outcomes on patient performance (e.g. gross motor, fine-motor), including socialization and communication skills some, such as bulbar function, are critical to ADLs and vital functions such as feeding and survival. INAD-RS is a combination of history and physical exam questions. In aggregate, the preponderance of the elements directly assess a patient’s 1) ability to carry on daily life activities (function) including those critical for survival, 2) ability to interact with their environment and caregivers, and 3) symptomatic status related to how they feel.

The INAD-RS is comprised of 40 items in six sub-categories of assessment, 1) Gross Motor Skills (24 points total), 2) Fine Motor Skills (12 points total), 3) Bulbar Function (14 points total), 4) Ocular (10 points total), 5) Temporo-frontal (16 points total), and, 6) Autonomic (4 points total). Each skill assessment is scored 0, 1, 2; higher score correlates with better performance. Table 1 shows the items of the definitions of each level of score and comments for each item. A normal child would have score of 80, 2 on each function × 40 and as the disease progresses the score declines. Of note, particularly young children may not have reached all normal developmental milestones at time of assessment (e.g. standing unaided) and these factors should be considered.

**Table 1:** The infantile neuroaxonal rating scale with comments and instructions

Item name and score definitions		Comments, instructions
1.	**Gross Motor Skills**	Consider aids of two small blocks, a small handheld bell, small spoon, stuffed animal or other bright toy to test visual tracking
	1. Hold head upright against gravity while sitting • Child cannot hold head erect for at least 3 s without support (Score = 0) • Child holds head erect for at least 3 s without support (Score = 1) • Child holds head erect and steady for at least 15 s without support (Score = 2)	• Typically performed on exam table
	2. Roll over • Child does not roll front to back or back to front (Score = 0) • Child rolls front to back or back to front, but not both (Score = 1) • Child rolls front to back and back to front (Score = 2)	• Typically performed on exam table
	3. Sit with support • Child cannot sit with support (Score = 0) • Child tenses muscles in an effort to maintain sitting position (Score = 1) • Child sits with slight support for at least 30 s (Score = 2)	• Typically performed on exam table
	4. Sit without support • Child cannot sit without support (Score = 0) • Child sits without support for at least 5 s (Score = 1) • Child sits without proper support for at least 30 s (Score = 2)	• Typically performed on exam table
	5. Stand aided • Child cannot stand aided (Score = 0) • Child can stand aided (Score = 1) • Child raises self to a standing position, using a chair or other convenient object for support	
	6. Stand unaided a. Child cannot stand unaided (Score = 0) b. Child can stand alone for at least 3 s after you release his or her hands (Score = 1) c. Child comes to a standing position without using any support (Score = 2)	
	7. Does head lag with dynamic change of position? a. Child cannot hold head when raised from supine to sitting by pulling on the arms (Score = 0) b. Child has head lag when raised from supine to sitting by pulling on the arms (Score = 1) c. Child has no head lag when raised from supine to sitting by pulling on the arms (Score = 2)	• Have child lie on table
	8. Peripheral limb function: Hand a. Child has no hand grip and/or contractures (Score = 0) b. Child shows finger grip (pincer) (Score = 1) c. Child holds object in hand (Score = 2)	• Give child block or small toy
	9. Peripheral limb function: Feet a. Child has contractures of both feet (Score = 0) b. Child has pes equinus or pes cavus without contracture (Score = 1) c. Child has no foot deformity (Score = 2)	
	10. Crawling a. Child cannot crawl (Score = 0) b. Child moves from lying prone to being up on hands and knees (Score = 1) c. Child makes forward progress of at least 5 ft by crawling on hands and knees (Score = 2)	
	11. Walk aided a. Child cannot walk with support (Score = 0) b. Child walks with support by a person and initiates multiple steps (Score = 1) c. Child walks independently while using or holding onto support (Score = 2)	
	12. Walk unaided a. Child cannot walk without support (Score = 0) b. Child takes at least 3 steps without support, even if gait is stiff-legged and wobbly (Score = 1) c. Child takes at least 5 steps independently, displaying coordination and balance (Score = 2)	
2.	**Fine Motor Skills**	
	1) Reaches for objects • Child does not reach for an object (Score = 0) • Child extends one or both arms forward to reach object, but does not touch object (Score = 1) • Child extends one or both arms forward to reach object and touches object with any part of either hand (Score = 2)	• Ensure object is light (e.g. light-weight block, light-weight bell)
	2) Grasps small objects • Child cannot pick up block (Score = 0) • Child picks up block using one or both hands (Score = 1) • Child uses pad of his or her thumb and any fingertip to grasp block (Score = 2)	
	3) Picks up food or spoon • Child cannot pick up food pellet or spoon (Score = 0) • Child grasps food pellet or spoon, but does not bring it to his/her mouth (Score = 1) • Child grasps food pellet or spoon and brings it to his/her mouth (Score = 2)	• Use plastic spoon, put in child’s hand and see if brings to mouth
	4) Rings bell • Child does not reach for bell (Score = 0) • Child extends one or both arms forward to reach bell and touches bell with any part of either hand (Score = 1) • Child picks up bell and attempts to ring bell (Score = 2)	
	5) Transfer objects • Child does not grasp ring when handed (Score = 0) • Child uses at least one hand to grasp ring for at least 2 s (Score = 1) • Child grasps ring and transfers from hand to hand (Score = 2)	
	6) Place one block on another • Child does not attempt to place one block on another (Score = 0) • Child attempts to place one block on another, but is unsuccessful (Score = 1) • Child is successful to place one block on another (Score = 2)	
3.	**Bulbar Function**	
	1) Swallows saliva • Child drools most of the time, requiring bib or several shirt changes per day (Score = 0) • Child drools occasionally (does not require a bib or a shirt change) (Score = 1) • Child does not drool (Score = 2)	• History based
	2) Swallows pureed food • Child cannot eat pureed food (Score = 0) • Child can occasionally eat pureed food (Score = 1) • Child can eat pureed food with no problem (Score = 2)	• History based
	3) Swallows solid food (including soft foods) • Child cannot eat solid food (Score = 0) • Child can occasionally eat solid food (Score = 1) • Child can eat solid food with no problem (Score = 2)	• History based
	4) Bite strength • Absent (Score = 0) • Weak (Score = 1) • Strong (Score = 2)	• Can place gloved hand in mouth to assess bite strength • Alternatively can be history based by asking parents / caregivers
	5) Nourishes liquids by syringe or tube feeding • Syringe feeding or tube feeding only (Score = 0) • Syringe feeding or tube feeding most of the time or occasional (Score = 1) • No syringe or tube feeding (Score = 2)	
	6) Tube feeding • Permanent (Score = 0) • Occasional (Score = 1) • Never (Score = 2)	
	7) Upper Airway • Tracheotomy or CPAP support (Score = 0) • Child has sleep apnea (Score = 1) • Child has normal sleep respiration (score = 2)	
4.	**Ocular**	
	1. Nystagmus • Child has nystagmus most of the time (Score = 0) • Child has occasional nystagmus (Score = 1) • Child has no nystagmus (Score = 2)	• Observe at rest, some have constant nystagmus, some only brief when shifting gaze
	2. Strabismus^a^ • Severe (Score = 0) • Moderate (Score = 1) • Mild/No Strabismus (Score = 2)	• Severe Strabismus: Constant exotropia • Moderate Strabismus: Exotropia > 50% of the exam before dissociation, or Exotropia < 50% of the exam before dissociation • Mild Strabismus: No exotropia unless dissociated, recovers in > 5 s; no exotropia unless dissociated, recovers in 1–5 s; or no exotropia unless dissociated, recovers in < 1 s (phoria)
	3. Tracks human face • Child does not track human face (score = 0) • Child fixes gaze on a person for at least 2 s (score = 1) • Child turns head to follow a person through the room (score = 2)	• Asses if they track face from around 1 ft. away
	4. Tracks object • Child does not track an object (score = 0) • Child’s eyes follow an object that is moved horizontally or vertically (Score = 1) • Child’s eyes follow an object that is moved in a circular motion (Score = 2)	• Use small toy
	5. Optic atrophy/temporal pallor • Child has severe optic atrophy/temporal pallor (Score = 0) • Child has moderate optic atrophy/temporal pallor (Score = 1) • Child has mild or no optic atrophy/temporal pallor (Score = 2)	• Either by way of indirect fundoscopy or slit-lamp exam by pediatric ophthalmologist
5.	**Temporo-frontal**	
	1) Interacts with parents or examiner • Child does not interact with parent or examiner (Score = 0) • Child clearly responds to the person’s voice (Score = 1) • Child actively participates in at least one play routine (Score = 2)	• Helpful to have parents elicit emotional response to parental voice or movement
	2) Responds to verbal commands • Child does not respond to verbal commands (Score = 0) • Child stops reaching for objects in response to “no,” but does not respond in an appropriate manner to other requests (Score = 1) • Child responds in an appropriate manner to at least one spoken request more complex than “no” (does not need to complete task) (Score = 2)	
	3) Repeats simple sounds • Child does not repeat simple sounds (Score = 0) • Child repeats a single vocalization only (Score = 1) • Child repeats two different, distinct vocalizations (Score = 2)	
	4) Smiles • Child does not smile nor vocalize mood (Score = 0) • Child expresses at least one mood (Score = 1) • Child’s mood or focus can change in response to speaker’s attention (Score = 2)	
	5) What is the child’s affect? • Sad, distressed or crying a lot (Score = 0) • Neutral affect (Score = 1) • Happy, ebullient, or cooperative (Score = 2)	
	6) Speaks individual words • Child does not speak individual words (Score = 0) • Child imitates at least one word, even if imitation consists of vowels only (Score = 1) • Child uses at least one word to make wants known (Score = 2)	
	7) Puts words together • Child does not use words (Score = 0) • Child uses at least one word to make wants known (Score = 1) • Child produces at least one utterance that includes two or more words (Score = 2)	
	8) Point to objects in a book • Child does not attempt to point to an object in a book (Score = 0) • Child points to object in a book, but does not identify object that was named (Score = 1) • Child points to object in a book that was named (Score = 2)	
6.	**Autonomic Nervous System**	
	1) Constipation • Child has fewer than 2 bowel movements per week and is dependent on a laxative (Score = 0) • Child has 2 or more bowel movements per week and is dependent on a laxative (Score = 1) • Child has 2 or more bowel movements per week without a laxative (Score = 2)	• History based
	2) Urinary • Indwelling catheter or dependent upon catherization (Score = 0) • Catherization no more than once per day (Score = 1) • No catherization required (Score = 2)	• History based

## Results

In total, there were 40 subjects with molecularly confirmed INAD examined at a range of 10–94 months since symptom onset (mean 39.4 months, SD 22.76). The INAD-RS score range was 7–69 (mean 34.78, SD 16.01). Patients who fit the phenotype of atypical INAD (ANAD) were excluded from the analysis. We found a strong correlation between the scale score and months since symptom onset (*r*^2^ = 0.48). This was a statistically significant correlation (*p* = 6.7 × 10^− 7^). We found the months since symptom onset to be the most sensitive indicator of disease progression, more sensitive than absolute age because of the frequent delay in diagnosis. In our experience, the rare nature of INAD make it a challenging diagnosis, and families are often on a diagnostic odyssey despite parental and medical awareness the child is not developing normally. This delay between symptom onset and diagnosis is in line with other pediatric genetic diseases [2, 11, 13–19].

### Intra-rater reliability

In a subset of 19 patients, the same examiner performed INAD-RS 2–4 weeks apart to demonstrate validity and reproducibility of INAD-RS. We found no statistically significant difference between the two visits, which demonstrates strong intra-rater reliability of INAD-RS. Means were 35.79 and 36.1 respectively for the two visits with a *p*-value for a statistically significant difference calculated to be 0.95, thus the null hypothesis of a mean difference of zero stands (t-test: two-sample assuming unequal variances).

### Inter-rater reliability

The INAD-RS neurological examinations were performed by the same examiner, under identical circumstances for each examination when possible, and under well-lit conditions with video recording and description. In general, these videos were of high quality with clear documentation of which part of INAD-RS was being performed, and the score awarded per assessment. Some examples of video narration included text overlays on video, and verbal calling out of score after each assessment on the scale. The authors independently reviewed the videos and assigned scores to each patient, blinded to the score of the original examiner, and we found no statistically significant difference between our scores and the examiner scores in all six domains demonstrating strong inter-rater reliability (t-test: two-sample assuming unequal variances).

In addition, we have experience in training other pediatric neurologists in performing INAD-RS. To date we have trained ten clinicians on how to perform INAD-RS by way of video conferencing with an experienced examiner. All of these training sessions lasted less than 1 h, and averaged ~ 30 min, with all trained clinicians verbalizing confidence in their ability to perform INAD-RS. Subsequent real-time observation of a subset of these trained clinicians validated their competence in performing INAD-RS.

### Rate of progression

**Fig. 1:**
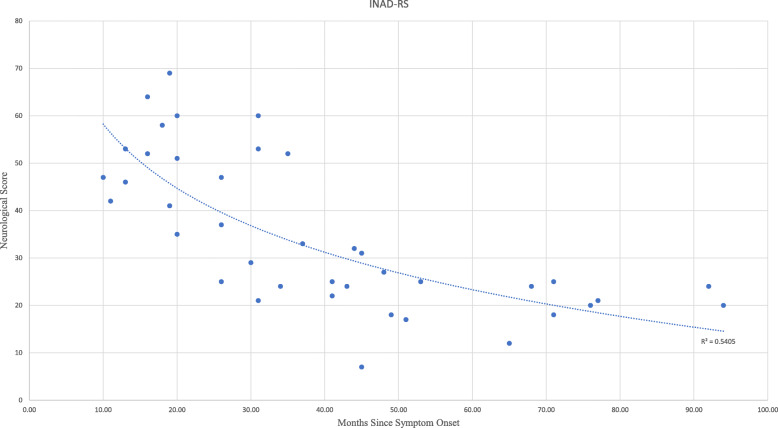
INAD-RS Progression. Figure showing months since symptom onset on X-axis and neurological score on Y-Axis. There is a negative correlation and an average loss of 0.49 points per month of symptoms. The best fit trend line is logarithmic (*r*2 = 0.54)

Rate of progression (see Fig. 1) on INAD-RS was calculated using a linear regression model applied to the cross-sectional data on the 40 subjects. The mean rate of progression was 0.49 points per month of symptoms (Standard error 00.08, lower 95% -0.658, upper 95% -0.324). There was no statistically significant difference between males and females. Interestingly, the best fit trend line is logarithmic (*r*^2^ = 0.54), and the linear trend line r^2^ is 0.48. The reason for a logarithmic slope more closely correlating with progression appears to be the increased loss of function earlier in the disease compared with later in the disease. Indeed, once many milestones have been lost, the residual function has less variance to ascertain differences. 60% of the maximum potential loss in the INAD-RS occurs within 40 months of onset of symptoms and this provides and optimum window for potential therapeutic intervention.

## Discussion

INAD-RS represents the first clinical outcome assessment derived specifically for INAD. The scale was created using the largest prospective cohort of INAD patients collected to date. The second largest cohort of 28 subjects, by Altuame et al. [11], consisted of a retrospective chart review and that study provided the most comprehensive overview of the natural history of INAD to our knowledge. We add to this literature by providing detailed disease progression data on a neurological scale tailored to INAD and documenting a pre-defined prospective exam on 40 patients.

One additional benefit of our scale is the ability to discern if a child is following the clinical course of INAD or atypical NAD (aNAD). While in general INAD presents sooner, some overlap exists and initially it can be challenging to differentiate between them. In some cases, the difference is only apparent as the disease progresses. In our experience monitoring a child longitudinally with INAD-RS using their months since symptom onset, it is quickly apparent if they are following a course of INAD or aNAD. To date, INAD-RS has not been used to assess aNAD or other PLAN disorders. We posit there may be a role for future studies to assess the utility of INAD-RS as a meaningful clinical outcome assessment in these and other conditions with a similar clinical phenotype.

Concerning limitations of INAD-RS, in general our experience with INAD-RS has shown us that it can be a convenient measure of clinical status and disability in INAD and be used as a meaningful tool to monitor progression. While we have demonstrated robust intra-rater and inter-rater reliability, some bias and difference in clinical experience will mean that scores will never correlate perfectly; however, this can be said of the majority of clinical assessment scales. INAD-RS also requires the examiner to be skilled and experienced in clinical examination of pediatric neurology and some of the markers require prior knowledge of normal clinical signs in this population.

Another limitation is these data represent a cross-sectional aggregate, and as such additional longitudinal data on each patient will be useful to monitor individual patient progression, and to look for other variables influencing progression, such as underlying genotype. The presence of a single truncating variant has previously been shown to be associated with more severe progression with a statistically significant difference [11]. Specifically Altuame et al. noted a statistical significant difference with the presence of a truncating variant in the time of initial concern (*p* = 0.04), initial loss of language (*p* = 0.001), initial loss of fine motor skills (*p* = 0.009), and initial loss of bulbar skills (*p* = 0.007). These significant differences were not observed in this cohort based on baseline cross-sectional analysis; however longitudinal monitoring which includes rate of progression to assess whether underlying genotype is affecting the phenotype should be performed at a future date to confirm this prior observation. Mechanistically regarding genotype-phenotype correlations in biochemical genetics, more severe mutations tend to result in less functional enzyme and thus infer a more severe phenotype; this is observed in other genetic disorders of enzymatic function and metabolism [2, 20–24].

## Conclusions

In conclusion, we report on a novel clinical outcome assessment tool for INAD. INAD-RS demonstrates a strong correlation between the scale score and months since symptom onset (logarithmic *r*^2^ = 0.54). There is a statistically significant correlation between the INAD-RS score and months since symptom onset (*p* = 6.7 × 10^− 7^). This scale assesses all six major domains of neurological sequelae in INAD, and thus assess a much more holistic view of INAD than the other scales (e.g. CHOP-INTEND, Hammersmith, Modified Ashworth). We are confident that we have developed and validated a novel neurological assessment score in INAD that demonstrates strong correlation with disease severity and disease progression. Given this strong correlation, we posit that INAD-RS would be suitable for a primary endpoint measure of disease monitoring and/or progression.

## Supplementary information

**Additional file 1: Supplementary Document 1.** INAD Neurological Assessment Instruction Manual.

**Additional file 2: Supplementary Document 2.** INAD Rating Scale Development Scoring Table.

## Data Availability

The authors confirm that the data supporting the findings of this study are available within the article [and/or] its supplementary materials.

## Abbreviations

aNAD: Atypical infantile neuroaxonal dystrophy
COA: Clinical outcome assessment
INAD: Infantile neuroaxonal dystrophy
INAD-RS: Infantile neuroaxonal dystrophy rating scale
PLAN: *PLA2G6*-associated neurodegeneration
