# Supplementary material for: The infantile neuroaxonal dystrophy rating scale (INAD-RS)
Source: Orphanet J Rare Dis. 2020 Jul 29;15:195. doi: 10.1186/s13023-020-01479-5 (PMC7392694; doi:10.1186/s13023-020-01479-5)
Supplement: Supplementary file 1 — Additional file 1: Supplementary Document 1. INAD Neurological Assessment Instruction Manual. [file 13023_2020_1479_MOESM1_ESM.docx]

**Supplementary Document 1: INAD Neurological Assessment Instruction Manual**

**Background, Indication & Purpose**

- For use in patients diagnosed with infantile neuroaxonal dystrophy to assess current functional status, burden of disease, and monitor disease progression longitudinally
- Infantile Neuroaxonal Dystrophy (INAD) is an extremely rare orphan disease affecting the axons, the part of the nerve cell that carries messages from the brain to other parts of the body. It is an inherited, autosomal recessive disease caused by changes in the PLA2G6 gene. PLA2G6 signals the body’s cells to make the enzyme A2 phospholipase, allowing for the breakdown of phospholipids in the cells. As the disease progresses, this abnormality in the housekeeping enzyme results in the accumulation of metabolic by-products ultimately affecting the integrity of the cell. Once overloaded, the cell dies. With the senescence of more cells, symptoms may start to appear as early as 3-6 months of age through about 3 years of age.
- Onset of symptoms are usually observed within the first 2 years of life. The typical progression in INAD is the loss of previously acquired skills such as the ability to sit, stand and vocalize. This progressive deterioration of movement, cognition and vision will continue unabated. Speech issues will likely be observed as well as difficulty with swallowing and chewing. Children with INAD also present with excessive drooling and can have choking or nasal regurgitation.
- Most children with the disease will deteriorate rapidly. Death usually occurs before puberty. These children are prone to secondary problems such as aspiration pneumonia or other infections. At present, there are no approved treatments that can stop the progression of the disease.

**General Information**

- Total possible score is 80, higher score indicated more function
- There are six sub-categories of assessment:
  - Gross Motor Skills (24 points total)
  - Fine Motor Skills (12 points total)
  - Bulbar Function (14 points total)
  - Ocular (10 points total)
  - Temporo-frontal (16 points total)
  - Autonomic (4 points total)
- Each skill assessment is scored 0, 1, 2; higher score correlates with better performance
  - If patient cannot perform test, appropriate score is 0

**Instructions**

- Administer INAD scale items in the order listed. Record performance in each category after each subscale exam. Do not go back and change scores.
- Follow directions provided for each exam technique. Scores should reflect what the patient does, not what the clinician thinks the patient can do. The clinician should record answers while administering the exam and work quickly.
- Infants should be assessed in diaper / onesie only unless infant is cold
- Ideally test first thing in AM or same time of day about 1 hour after feeding
- Test should be performed on appropriate clinical examination table or firm padded mat
- Consider examination aids of two small blocks, a small handheld bell, small spoon, stuffed animal or other bright toy to test visual tracking
