# Supplementary material for: The infantile neuroaxonal dystrophy rating scale (INAD-RS)
Source: Orphanet J Rare Dis. 2020 Jul 29;15:195. doi: 10.1186/s13023-020-01479-5 (PMC7392694; doi:10.1186/s13023-020-01479-5)
Supplement: Supplementary file 2 — Additional file 2: Supplementary Document 2. INAD Rating Scale Development Scoring Table. [file 13023_2020_1479_MOESM2_ESM.docx]

| Gross Motor Skill | Score | Source |
| --- | --- | --- |
| Hold head upright against gravity while sitting | Child cannot hold head erect for at least 3 seconds without support (Score = 0) | Child cannot do skill to get score 1 or 2 |
|  | Child holds head erect for at least 3 seconds without support (Score = 1) | Bayley Motor Scale – Gross Motor Subtest  Item #4 - Controls Head While Upright Series: 3 seconds  *Child holds head erect for at least 3 seconds without support* |
|  | Child holds head erect and steady for at least 15 seconds without support (Score = 2) | Bayley Motor Scale – Gross Motor Subtest  Item #9 - Controls Head While Upright Series: 15 seconds  *Child holds head erect for at least 15 seconds without support* |
| Roll over | Child does not roll front to back or back to front (Score = 0) | Child cannot do skill to get score 1 or 2 |
|  | Child rolls front to back or back to front, but not both (Score = 1) | Bayley Motor Scale – Gross Motor Subtest  Item #25 – Rolls from Back to Stomach  *Child rolls from back to stomach, rolling from either side* |
|  | Child rolls front to back and back to front (Score = 2) | Based on video observation of children with INAD |
| Sit with support | Child cannot sit with support (Score = 0) | Child cannot do skill to get score 1 or 2 |
|  | Child tenses muscles in an effort to maintain sitting position (Score = 1) | Bayley Motor Scale – Gross Motor Subtest  Item # 16 – Sits with Support Series: Briefly  *Child tenses muscles in an effort to maintain sitting position* |
|  | Child sits with slight support for at least 30 seconds (Score = 2) | Bayley Motor Scale – Gross Motor Subtest  Item # 19 – Sits with Support Series: 30 seconds  *Child sits with slight support for at least 30 seconds* |
| Sit without support | Child cannot sit without support (Score =0) | Child cannot do skill to get score 1 or 2 |
|  | Child sits without support for at least 5 seconds (Score = 1) | Bayley Motor Scale – Gross Motor Subtest  Item # 19 – Sits without Support Series: 5 seconds  *Child sits alone without support for at least 5 seconds* |
|  | Child sits without proper support for at least 30 seconds (Score = 2) | Bayley Motor Scale – Gross Motor Subtest  Item # 26 – Sits without Support Series: 30 seconds  *Child sits alone without support for at least 30 seconds* |
| Stand aided | Child cannot stand aided (Score = 0) | Child cannot do skill to get score 1 or 2 |
|  | Child can stand aided (Score = 1) | Based on video observation of children with INAD |
|  | Child raises self to a standing position, using a chair or other convenient object for support (Score = 2) | Bayley Motor Scale – Gross Motor Subtest  Item # 35 – Raises Self to Standing Position  *Child raises self to a standing position, using a chair or other convenient object for support* |
| Stand unaided | Child cannot stand unaided (Score = 0) | Child cannot do skill to get score 1 or 2 |
|  | Child can stand alone for at least 3 seconds after you release his or her hands (Score = 1) | Bayley Motor Scale – Gross Motor Subtest  Item # 40 – Stands Alone  *Child stands alone for at least 3 seconds after you release his or her hands* |
|  | Child comes to a standing position without using any support (Score = 2) | Bayley Motor Scale – Gross Motor Subtest  Item # 41 – Stand Up Series: Alone  *Child comes to a standing position, rolling first to a prone or quadruped position, without using any support* |
| Does head lag with dynamic change of position? | Child cannot hold head when raised from supine to sitting by pulling on the arms (Score = 0) | Child cannot do skill to get score 1 or 2 |
|  | Child has head lag when raised from supine to sitting by pulling on the arms (Score = 1) | Based on video observation of children with INAD |
|  | Child has no head lag when raised from supine to sitting by pulling on the arms (Score = 2) | Based on video observation of children with INAD |
| Peripheral limb function: Hand | Child has no hand grip and/or contractures (Score = 0) | Child cannot do skill to get score 1 or 2 |
|  | Child shows finger grip (pincer) (Score = 1) | Based on video observation of children with INAD |
|  | Child holds object in hand (Score = 2) | Based on video observation of children with INAD |
| Peripheral limb function: Feet | Child has contractures of both feet (Score = 0) | Child cannot do skill to get score 1 or 2 |
|  | Child has pes equinus or pes cavus without contracture (Score = 1) | Based on video observation of children with INAD |
|  | Child has no foot deformity (Score = 2) | Based on video observation of children with INAD |

| Crawling | Child cannot crawl (Score = 0) | Child cannot do skill to get score 1 or 2 |
| --- | --- | --- |
|  | Child moves from lying prone to being up on hands and knees (Score = 1) | Bayley Motor Scale – Gross Motor Subtest  Item # 31 – Crawl Series: Crawl Position  *Child moves from lying prone to being up on hands and knees* |
|  | Child makes forward progress of at least 5 feet by crawling on hands and knees (Score = 2) | Bayley Motor Scale – Gross Motor Subtest  Item # 34 – Crawl Series: Crawl Movement  *Child makes forward progress of at least 5 feet by crawling on hands and knees* |
| Walk aided | Child cannot walk with support (Score = 0) | Child cannot do skill to get score 1 or 2 |
|  | Child walks with support by a person and initiates multiple steps (Score = 1) | Bayley Motor Scale – Gross Motor Subtest  Combination of Item # 29 and Item # 37 with the support being a person  Item # 29 – Makes Stepping Movements  *Child makes at least two stepping movements that propel him or her forward, even if child does not fully support own weight*  AND  Item # 37 – Walks Series: With Support  *Child walks by making coordinated, alternating stepping movements* |
|  | Child walks independently while using or holding onto support (Score = 2) | Bayley Motor Scale – Gross Motor Subtest  Combination of Item # 37 and Item # 38 with the support being not a person  Item # 37 – Walks Series: With Support  *Child walks by making coordinated, alternating stepping movements*  AND  Item # 38 – Walks Sideways with Support  *Child walks sideways while holding onto furniture for support and balance* |

| Walk unaided | Child cannot walk without support (Score = 0) | Child cannot do skill to get score 1 or 2 |
| --- | --- | --- |
|  | Child takes at least 3 steps without support, even if gait is stiff-legged and wobbly (Score = 1) | Bayley Motor Scale – Gross Motor Subtest  Item # 42 – Walks Series: Alone  *Child takes at least 3 steps without support, even if gait is stiff-legged and wobbly* |
|  | Child takes at least 5 steps independently, displaying coordination and balance (Score = 2) | Bayley Motor Scale – Gross Motor Subtest  Item # 43 – Walks Series: Alone with Coordination  *Child takes at least 5 steps independently, displaying coordination and balance* |

| Fine Motor Skills | Score | Source |
| --- | --- | --- |
| Reaches for objects | Child does not reach for an object (Score = 0) | Child cannot do skill to get score 1 or 2 |
|  | Child extends one or both arms forward to reach object, but does not touch object (Score = 1) | Bayley Motor Scale – Fine Motor Subtest  Item # 13 – Block Series: Reaches for Block  *Child extends one or both arms forward to reach object, but does not touch object* |
|  | Child extends one or both arms forward to reach object, and touches object with any part of either hand (Score = 2) | Bayley Motor Scale – Fine Motor Subtest  Item # 14 – Block Series: Touches Block  *Child extends one or both arms forward to reach object, and touches object with any part of either hand* |
| Grasps small objects | Child cannot pick up block (Score = 0) | Child cannot do skill to get score 1 or 2 |
|  | Child picks up block using one or both hands (Score = 1) | Bayley Motor Scale – Fine Motor Subtest  Item # 15 – Block Series: Whole Hand Grasp  *Child picks up block using one or both hands* |
|  | Child uses pad of his or her thumb and any fingertip to grasp block (Score = 2) | Bayley Motor Scale – Fine Motor Subtest  Item # 22 – Block Series: Thumb-Fingertip Grasp  *Child uses pad of his or her thumb and any fingertip to grasp block* |
| Picks up food or spoon | Child cannot pick up food pellet or spoon  (Score = 0) | Child cannot do skill to get score 1 or 2 |
|  | Child grasps food pellet or spoon, but does not bring it to his/her mouth (Score = 1) | Based on video observation of children with INAD |
|  | Child grasps food pellet or spoon and brings it to his/her mouth (Score = 2) | Based on video observation of children with INAD |
| Rings bell | Child does not reach for bell (Score = 0) | Child cannot do skill to get score 1 or 2 |
|  | Child extends one or both arms forward to reach bell, and touches bell with any part of either hand (Score = 1) | Based on video observation of children with INAD |
|  | Child picks up bell and attempt to ring bell  (Score = 2) | Based on video observation of children with INAD |

| Transfer objects | Child does not grasp ring when handed  (Score = 0) | Child cannot do skill to get score 1 or 2 |
| --- | --- | --- |
|  | Child uses at least one hand to grasp ring for at least 2 seconds (Score = 1) | Bayley Motor Scale – Fine Motor Subtest  Item # 6 – Retains Ring  *Child retains ring for at least 2 seconds* |
|  | Child grasps ring and transfer from hand to hand (Score = 2) | Bayley Motor Scale – Fine Motor Subtest  Item # 19 – Transfers Ring  Child transfers from hand to hand |
| Place one block on another | Child does not attempt to place one block on another (Score = 0) | Child cannot do skill to get score 1 or 2 |
|  | Child attempts to place one block on another, but is unsuccessful (Score = 1) | Bayley Motor Scale – Fine Motor Subtest  Item # 31 – Block stacking series: 2 Blocks  Child Stacks at least 2 blocks |
|  | Child is successful to place one block on another (Score = 2) | Bayley Motor Scale – Fine Motor Subtest  Item # 31 – Block stacking series: 2 Blocks  Child Stacks at least 2 blocks |

| Bulbar Function | Score | Source |
| --- | --- | --- |
| Swallows saliva | Child drools most of the time, requiring bib or several shirt changes per day (Score = 0) | Based on video observation of children with INAD |
|  | Child drools occasionally (does not require a bib or a shirt change) (Score = 1) | Based on video observation of children with INAD |
|  | Child does not drool (Score = 2) | Based on video observation of children with INAD |
| Swallows pureed food | Child cannot eat pureed food (Score = 0) | Based on interviews with parents of INAD children |
|  | Child can occasionally eat pureed food  (Score = 1) | Based on interviews with parents of INAD children |
|  | Child can eat pureed food with no problem (Score = 2) | Based on interviews with parents of INAD children |
| Swallows solid food (including soft foods) | Child cannot eat solid food (Score = 0) | Based on interviews with parents of INAD children |
|  | Child can occasionally eat solid food (Score = 1) | Based on interviews with parents of INAD children |
|  | Child can eat solid food with no problem  (Score = 2) | Based on interviews with parents of INAD children |
| Bite strength | Absent (Score = 0) | Based on interviews with parents of INAD children |
|  | Weak (Score = 1) | Based on interviews with parents of INAD children |
|  | Strong (Score = 2) | Based on interviews with parents of INAD children |
| Nourishes liquids by syringe or tube feeding | Syringe feeding or tube feeding only (Score = 0) | Based on interviews with parents of INAD children |
|  | Syringe feeding or tube feeding most of the time or occasional (Score = 1) | Based on interviews with parents of INAD children |
|  | No syringe or tube feeding (Score = 2) | Based on interviews with parents of INAD children |
| Tube feeding | Permanent (Score = 0) | Based on interviews with parents of INAD children |
|  | Occasional (Score = 1) | Based on interviews with parents of INAD children |
|  | Never (Score = 2) | Based on interviews with parents of INAD children |
| Upper Airway | Tracheotomy or CPAP support (Score = 0) | Based on interviews with parents of INAD children |
|  | Child has sleep apnea (Score = 1) | Based on interviews with parents of INAD children |
|  | Child has normal sleep respiration (score = 2) | Based on interviews with parents of INAD children |

| Ocular | Score | Source |
| --- | --- | --- |
| Nystagmus | Child has nystagmus most of the time (Score = 0) | Based on video observation of children with INAD and input from KOL |
|  | Child has occasional nystagmus (Score = 1) | Based on video observation of children with INAD and input from KOL |
|  | Child has no nystagmus (Score = 2) | Based on video observation of children with INAD and input from KOL |
| Strabismus | Severe (Score = 0) | Severe Strabismus: Constant exotropia |
|  | Moderate (Score = 1) | Moderate Strabismus: Exotropia > 50 % of the exam before dissociation, or Exotropia < 50 % of the exam before dissociation |
|  | Mild/No Strabismus (Score = 2) | Mild Strabismus: No exotropia unless dissociated, recovers in > 5 seconds, no exotropia unless dissociated, recovers in 1-5 seconds, or no exotropia unless dissociated, recovers in < 1 second (phoria) |
| Tracks human face | Child does not track human face (score = 0) | Child cannot do skill to get score 1 or 2 |
|  | Child fixes gaze on a person for at least 2 seconds (score = 1) | Based on video observation of children with INAD and Bayley Motor Scale – Language Scale: Receptive Communication Subtest  Item # 1 – Regards Person Momentarily  Child fixes gaze on a person for at least 2 seconds |
|  | Child turns head to follow a person through the room (score = 2) | Based on video observation of children with INAD |
| Tracks object | Child does not track an object (score = 0) | Based on video observation of children with INAD and input from KOL |
|  | Child’s eyes follow an object that is moved horizontally or vertically (Score = 1) | Based on video observation of children with INAD and input from KOL |
|  | Child’s eyes follow an object that is moved in a circular motion (Score = 2) | Based on video observation of children with INAD and input from KOL |

| Optic atrophy/temporal pallor | Child has severe optic atrophy/temporal pallor (Score = 0) | Based on input from KOL |
| --- | --- | --- |
|  | Child has moderate optic atrophy/temporal pallor (Score = 1) | Based on input from KOL |
|  | Child has mild or no optic atrophy/temporal pallor (Score = 2) | Based on input from KOL |

| Temporo-frontal | Score | Source |
| --- | --- | --- |
| Interacts with parents or examiner | Child does not interact with parent or examiner (Score = 0) | Based on video observation of children with INAD, interview of parents and input from KOL |
|  | Child clearly responds to the person’s voice (Score = 1) | Based on video observation of children with INAD, interview of parents and input from KOL |
|  | Child actively participates in at least one play routine (Score = 2) | Based on video observation of children with INAD, interview of parents and input from KOL |
| Responds to verbal commands | Child does not respond to verbal comments (Score = 0) | Based on video observation of children with INAD, interview of parents and input from KOL |
|  | Child stops reaching for objects in response to “no”, and does not respond in an appropriate manner to other requests (Score = 1) | Based on video observation of children with INAD, interview of parents and input from KOL |
|  | Child responds in an appropriate manner to at least one spoken request more complex than “no” (does not need to complete task) (Score = 2) | Based on video observation of children with INAD, interview of parents and input from KOL |
| Repeats simple sounds | Child does not repeat simple sounds (Score = 0) | Based on video observation of children with INAD, interview of parents and input from KOL |
|  | Child repeats a single vocalization only (Score = 1) | Based on video observation of children with INAD, interview of parents and input from KOL |
|  | Child repeats two different, distinct vocalizations (Score = 2) | Based on video observation of children with INAD, interview of parents and input from KOL |
| Smiles | Child does not smile nor vocalize mood (Score = 0) | Based on video observation of children with INAD, interview of parents and input from KOL |
|  | Child expresses at least one mood (Score = 1) | Based on video observation of children with INAD, interview of parents and input from KOL |
|  | Child‘s mood or focus can change in response to speaker’s attention (Score = 2) | Based on video observation of children with INAD, interview of parents and input from KOL |
| What is the child’s affect? | Sad, distressed or crying a lot (Score = 0) | Based on video observation of children with INAD, interview of parents and input from KOL |
|  | Neutral affect (Score =1) | Based on video observation of children with INAD, interview of parents and input from KOL |
|  | Happy, ebullient, or cooperative (Score = 2) | Based on video observation of children with INAD, interview of parents and input from KOL |
| Speaks individual words | Child does not speak individual words (Score = 0) | Based on video observation of children with INAD, interview of parents and input from KOL |
|  | Child imitates at least one word, even if imitation consists of vowels only (Score = 1) | Based on video observation of children with INAD, interview of parents and input from KOL |
|  | Child uses at least one word to make wants known (Score = 2) | Based on video observation of children with INAD, interview of parents and input from KOL |
| Puts words together | Child does not use words (Score = 0) | Based on video observation of children with INAD, interview of parents and input from KOL |
|  | Child uses at least one word to make wants known (Score = 1) | Based on video observation of children with INAD, interview of parents and input from KOL |
|  | Child produces at least one utterance that includes two or more words (Score = 2) | Based on video observation of children with INAD, interview of parents and input from KOL |
| Point to objects in a book | Child does not attempt to point to an object in a book (Score = 0) | Based on video observation of children with INAD, interview of parents and input from KOL |
|  | Child points to object in a book, but does not identify object that was named (Score = 1) | Based on video observation of children with INAD, interview of parents and input from KOL |
|  | Child points to object in a book that was named (Score = 2) | Based on video observation of children with INAD, interview of parents and input from KOL |

| Autonomic Nervous System | Score | Source |
| --- | --- | --- |
| Constipation | Child has fewer than 2 bowel movement per week and is dependent on a laxative (Score = 0) | Based on interview of parents of INAD children and input from KOL |
|  | Child has 2 or more bowel movements per week and is on a laxative (Score = 1) | Based on interview of parents of INAD children and input from KOL |
|  | Child has 2 or more bowel movements per week without a laxative (Score = 2) | Based on interview of parents of INAD children and input from KOL |
| Urinary | Indwelling catheter or dependent upon catherization (Score = 0) | Based on interview of parents of INAD children and input from KOL |
|  | Catherization no more than once per day  (Score = 1) | Based on interview of parents of INAD children and input from KOL |
|  | No catherization required (Score = 2) | Based on interview of parents of INAD children and input from KOL |

Abbreviations: INAD – Infantile Neuroaxonal dystrophy; KOL – Key opinion leader;
